# Gastrointestinal cancer organoids—applications in basic and translational cancer research

**DOI:** 10.1038/s12276-021-00654-3

**Published:** 2021-10-18

**Authors:** Therese Seidlitz, Daniel E. Stange

**Affiliations:** 1 Department of Visceral, Thoracic and Vascular Surgery, Medical Faculty and University Hospital Carl Gustav Carus, Technische Universität Dresden, Dresden, Germany; 2 National Center for Tumor Diseases (NCT), Dresden, Germany: German Cancer Research Center (DKFZ), Heidelberg, Germany; Helmholtz-Zentrum Dresden - Rossendorf (HZDR), Dresden, Germany

**Keywords:** Gastrointestinal cancer, Cancer models

## Abstract

Cancer is a major health problem and a leading cause of death worldwide. Early cancer detection and continuous changes in treatment strategies have improved overall patient survival. The recent development of targeted drugs offers new opportunities for personalized cancer treatment. Nevertheless, individualized treatment is accompanied by the need for biomarkers predicting the response of a patient to a certain drug. One of the most promising breakthroughs in recent years that might help to overcome this problem is the organoid technology. Organoid cultures exhibit self-renewal capacity, self-organization, and long-term proliferation, while recapitulating many aspects of their primary tissue. Generated patient-derived organoid (PDO) libraries constitute “living” biobanks, allowing the in-depth analysis of tissue function, development, tumor initiation, and cancer pathobiology. Organoids can be derived from all gastrointestinal tissues, including esophageal, gastric, liver, pancreatic, small intestinal and colorectal tissues, and cancers of these tissues. PDOs are amenable to various techniques, including sequencing analyses, drug screening, targeted therapy testing, tumor microenvironment studies, and genetic engineering capabilities. In this review, we discuss the different applications of gastrointestinal organoids in basic cancer biology and clinical translation.

## Cancer: personalized models of gastrointestinal tumors

Three-dimensional mini-organs or “organoids” derived from patients with gastrointestinal tumors can offer new insights into cancer biology and guide the selection of appropriate therapies. By cultivating gastrointestinal tumor cells with an appropriate combination of growth factors, researchers can generate organoids that recapitulate key features of the original malignancy in miniature. Therese Seidlitz and Daniel E. Stange from the University Hospital Carl Gustav Carus in Dresden, Germany have reviewed the use of such models for cancer research and clinical care. These organoids can be used to identify the treatment strategies most likely to be effective in a given patient, or to assess differences between primary tumors and their metastases. Researchers can also genetically manipulate organoids made from healthy tissue to study the biological pathways that contribute to tumor development and disease progression in cancer patients.

## Introduction

In recent decades, our knowledge of the origin and progression of cancer has increased tremendously. Early cancer detection and improved standard-of-care cancer treatments have increased overall survival. In addition, the development of targeted therapies based on the individual genetic alterations of a patient’s tumor offers new opportunities. Despite the improved understanding, cancer remains a major worldwide health problem ranking as the leading cause of death^1^. One of the most important challenges for further improvement of cancer therapy is the translation of achieved results from bench to bedside. A plethora of results are still generated in cancer models that poorly recapitulate the behavior of patients’ tumors. This often leads to poor reproducibility in the clinic. An example is the extraordinarily high number of newly developed drugs that fail in clinical trials but perform well in cancer models^2^. Immortalized traditional two-dimensional (2D) cancer cell lines are still the backbone of current human cancer research. These cancer cell lines, which are characterized by low handling costs and ease of use, mostly do not reflect the parental tumor due to the outgrowth of only a few cancer cells during cell line establishment and the extensive adaption to the growth conditions on cell culture plates. Regular passaging, often over many decades, leads to monogenetic cell lines that do not recapitulate the heterogeneity of the tumors they were derived from. In contrast, patient-derived tumor xenograft (PDTx) models maintain the histology and genomic aberration patterns of their parental tumors to a much greater degree. Their use in cancer research is nevertheless restricted due to their high costs and the length of time needed for their establishment, usually months.

Recently developed three-dimensional culture systems termed organoids open up new opportunities in cancer research, including efforts to personalize cancer treatment. Organoids have been shown to retain the heterogeneity and genetic identity of the cancer tissue from which they are derived. The generation of cancer organoids is accomplished in a short time (weeks), often with high efficiency, and cultures can usually be passaged an unlimited number of times. Even though they are still expensive due to the complex growth factor cocktail in the medium and the necessity for an extracellular matrix (ECM), organoid cultures are much cheaper than mouse-based PDTx models. The recently reported development of large “living” biobanks of patient-derived cancer organoid (PDO) libraries reveals opportunities for the in-depth analysis of cancer biology as well as translational clinical applications. In this review, we describe different gastrointestinal organoid methodologies and their applications for basic and translational cancer research.

## Development of gastrointestinal organoids

Organoids can be derived out of primary tissue from adult stem cells (AdSCs) or from embryonic stem cells and induced pluripotent stem cells (together PSCs)^3,4^. AdSCs can only generate cells related to the tissue from which they are derived, requiring a growth factor enriched medium that mimics the stem cell niche of the original tissue. In contrast, PSCs have the ability to differentiate into any cell of the human body if a stepwise differentiation protocol has been established. Another difference is the time period until the culture is established: while AdSC-derived organoids can be set up in a short period (usually a few weeks), establishing PSC-derived organoids takes ~20–60 days depending on the complexity of the differentiation protocol. Nevertheless, the main difference is related to the tissue type, while AdSC-derived organoids contain cells from only one tissue type (e.g., epithelial cells), PSC-derived organoids can contain cells of multiple tissue types (e.g., epithelial and mesenchymal cells).

**Fig. 1:**
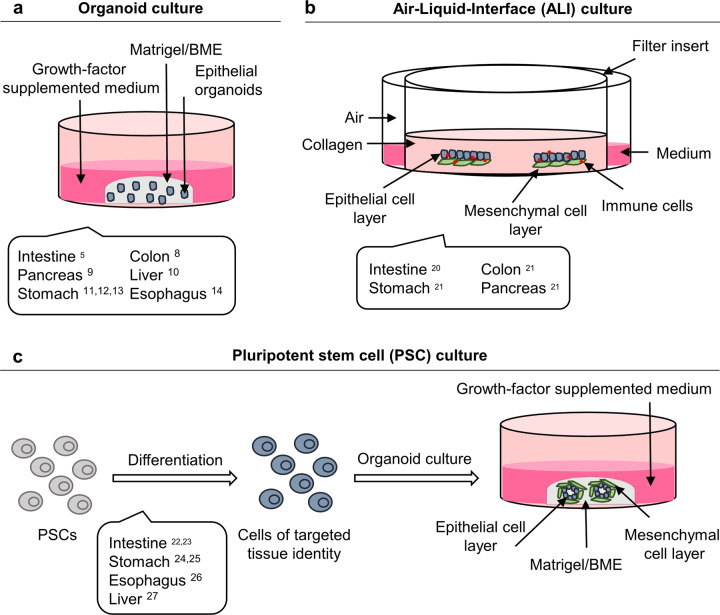
Organoid culture methods. **a** Adult stem cell-derived epithelial organoids are embedded in a laminin-rich extracellular matrix-like (Matrigel^®^ or BME^®^) and overlaid with growth factor-supplemented medium. **b** In the air-liquid-interface (ALI) organoid cultivation approach, epithelial tissue is cultivated with the surrounding stroma, including fibroblasts and immune cells. The tissue is grown in a type I collagen matrix on top of a transwell insert containing a permeable membrane providing the cells with growth medium. **c** Pluripotent stem cell (PSC)-derived organoids are generated by a stepwise differentiation protocol that guides PSCs into the tissue of interest. PSC-derived organoids are also embedded in Matrigel^®^ or BME^®^ and overlaid with growth factor-supplemented medium.

In 2009, Sato et al. first described the cultivation of mouse small intestinal organoids established from intestinal crypts or directly from leucine-rich repeat-containing G-protein coupled receptor 5 (LGR4/5/6)-expressing intestinal stem cells^5^. To achieve this, detailed knowledge about the signaling pathways active in the intestinal stem cell niche was applied. After embedding in Matrigel^®^, intestinal crypts were cultured in serum-free medium containing the following growth factors: Rspondin (an LGR5 ligand and WNT signaling agonist^6,7^), epidermal growth factor (EGF) and Noggin (a bone morphogenetic protein inhibitor). In the case of initiation from Lgr5^+^ intestinal stem cells, WNT was needed in the medium for the first days until Paneth cells had formed. The organoids grew as highly polarized epithelial structures with proliferative crypts and differentiated villus compartments^5^. The established protocol was later adopted to develop organoids from murine and human gastrointestinal epithelial tissues, including those of the colon^8^, pancreas^9^, liver^10^, stomach^11–13^, and esophagus^14^. Generally, AdSC-derived organoids are embedded in a laminin-rich ECM (such as Matrigel^®^ or Basement Membrane Extract (BME^®^)), overlaid with growth factor-supplemented medium to maintain the stem cell niche, which allows self-renewal and long-term proliferation (Fig. 1a). Organoids can be expanded to generate more cellular mass and can also be cryopreserved and genetically modified. They typically reflect the physiological functions and histology of the parental tissues^4,15–18^. An important feature of organoid cultures derived from non-tumorous tissue is their genomic stability over a long period of time in culture^10,19^.

In 2009, the Kuo laboratory described a different cultivation approach for murine intestinal epithelial organoids by using an air-liquid interface (ALI) system^20^. With this system, the epithelial cells as well as the surrounding stroma, including fibroblasts and immune cells, are cultivated together, representing a more organotypic approach (Fig. 1b). The mechanically dissociated tissue is grown in a type I collagen matrix on top of an inner Transwell insert. The latter contains a permeable membrane providing the cells with growth medium. The surrounding stromal cells are sufficient to facilitate organoid growth with no further addition of tissue-specific growth factors. By using this method, intestinal, colon, stomach, and pancreas organoids comprising epithelial and mesenchymal cells could be cultured, allowing proliferation and lineage differentiation^20,21^. PSC-derived organoids have also been described for different gastrointestinal tissues, e.g., the small and large intestine^22,23^, stomach^24,25^, esophagus^26^, and liver^27^. As opposed to AdSC-derived organoids, a specific stepwise differentiation protocol that guides PSCs into the tissue of interest is applied, finally allowing the full-grown organoids to be embedded in ECM and overlaid with growth factor-supplemented medium (Fig. 1c).

## Applications of gastrointestinal cancer organoids in cancer research

**Fig. 2:**
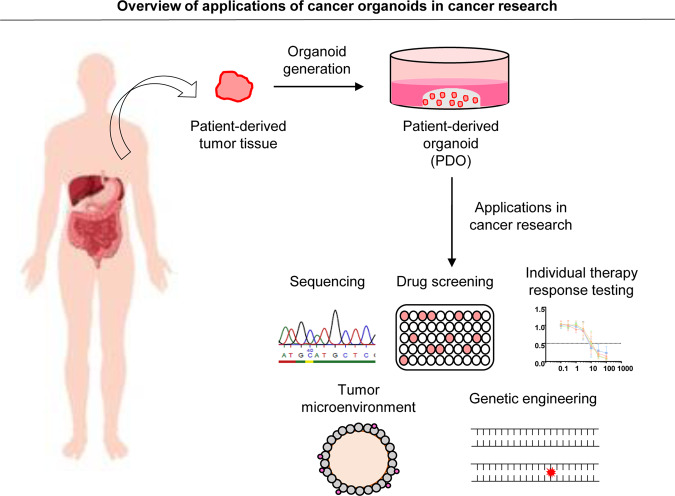
Applications of cancer organoids in cancer research. Generated patient-derived organoids (PDOs) from tumor biopsies have numerous applications in translational cancer research, including sequencing analyses, drug screenings, targeted therapy testings, tumor microenvironment studies and genetic engineering capabilities using CRISPR/Cas9 technology, and other applications.

Organoid models have also been described for numerous types of human cancers. In the following part of the review, we describe the different applications specifically for gastrointestinal PDOs. To date, PDO biobanks have been established within the gastrointestinal tract for colorectal cancer (CRC)^28–32^ and gastric cancers, including esophageal adenocarcinoma (GC/EAC)^33–40^, esophageal squamous cell carcinoma (ESCC)^41^, liver cancer (LC)^42–44^, pancreatic ductal adenocarcinoma (PDAC)^9,45–47^, and gastroenteropancreatic neuroendocrine neoplasms (GEP-NENs)^48^ (Fig. 2).

### Analysis of the mutational landscape using high-throughput sequencing technologies

**Fig. 3:**
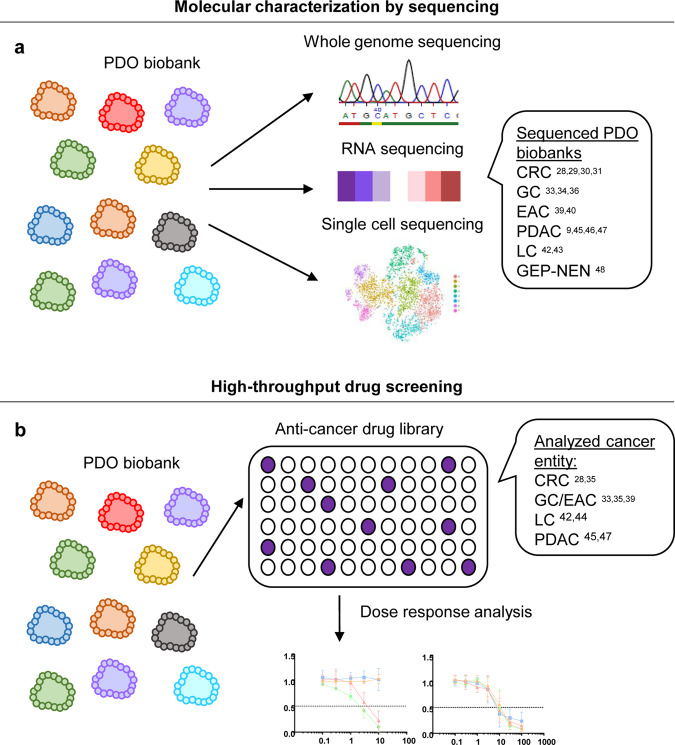
Molecular characterization and high-throughput drug screening using cancer organoids. **a** PDO biobanks for gastrointestinal cancer were characterized by whole-genome sequencing, RNA sequencing, and single-cell sequencing. **b** High-throughput drug screening assays on generated PDO biobanks using differently sized anticancer drug libraries have been reported, allowing dose response analyses of the PDOs.

PDOs derived from patient tumor material have extended our molecular understanding of human cancer. As nucleic acids can be easily retrieved in high quality and without contaminating nucleic acids from the microenvironment, characterization by sequencing can be performed in a straightforward manner without DNA/RNA amplification steps and sophisticated bioinformatic approaches to denoise the data from non-tumor-derived signals (Fig. 3a). Sequencing of PDOs can identify subclonal mutations present at low frequencies that were not detectable in primary tumor samples.

The first cancer organoid biobank generated comprised a set of 20 individual CRC PDOs^28^. In-depth DNA sequencing and RNA expression analysis showed that PDOs recapitulated the somatic copy number alterations (SCNA) as well as the mutation spectra found in CRC. Typical CRC inactivating mutations in the tumor suppressor genes *APC*, *TP53*, *FBXW7,* and *SMAD4* as well as activating mutations in *KRAS*, *PIK3CA*, *BRAF*, and *TGFBR1/2* were recognized. A separate study using a subset of these PDOs performed proteomic analyses, which proved that proteomic approaches in PDOs are feasible^49^. Distinct organoid profiles at the proteomic level were observed between the PDOs and matching healthy counterparts as well as between different patients, corroborating the importance of personalized profiling of each individual sample. In line with these studies, Fujii et al. generated an organoid biobank composed of 55 CRC PDOs of different histological subtypes and clinical statuses^29^. Genetic profiling of PDOs revealed the typical mutational spectrum of CRCs^50^. The authors revealed that individual PDO lines exhibited different growth factor dependencies on the basis of present or absent genetic alterations. As an example, all adenoma PDOs grew without WNT and Rspondin supplementation. Alterations in *Kras*^*G12V*^ led to EGF independence. Microsatellite-stable organoids were less dependent on Noggin, A83-01, and normoxic culture conditions. Yao et al. reported a biobank of 80 locally advanced rectal cancer PDOs from biopsy specimens^31^. Eighteen PDO lines were analyzed by whole-exome sequencing recapitulating the mutational profile observed in the matched tumor tissue. Known cancer driver mutations were found with a 94.4% overlap with the described TCGA dataset^50^. Most altered genes, including *APC*, *FBXW7*, *ARID1A*, *LRP5*, and *SOX9*, were related to the WNT pathway (88.9%). Ganesh et al. reported that 77% of mutations in primary rectal cancers could be detected in the corresponding PDOs^30^.

Several studies have reported the generation of GC PDO libraries^33–38^. Three focused on the molecular classification of gastric cancer and revealed that PDOs recapitulated the mutational pattern of their primary tumors^33,34,36^. GC can be molecularly divided into four subtypes: the chromosomal instability (CIN) subtype with frequent *TP53* mutations and RTK-RAS pathway amplifications; the genomically stable (GS) subtype displaying diffuse tumor morphology and *CDH1* as well as *RHOA* alterations; the microsatellite instability (MSI) subtype with a hypermutation phenotype; and the Epstein-Barr virus-positive (EBV) subtype showing frequent *PIK3CA* mutations and silencing of *CDKN2A*^51^. Of note, PDO cultures of all four GC subtypes could be established, indicating that the culture conditions do not exclude the successful outgrowth of certain subtypes^33,34,36^. Esophageal cancer is histologically classified as either EAC or ESCC. While EAC closely resembles the CIN subtype of GC with a high number of chromosomal aberrations, mRNA expression, DNA methylation, and SCNA data demonstrated that ESCC closely resembles head and neck squamous cell carcinoma at the molecular level^52^. Two studies validated the genomic landscape of primary EAC PDOs, which included the main cancer drivers *TP53*, *CDKN2A*, *KCNQ3*, *PIK3CA*, *MUC6*, and *ARID1B*^39,40^. RNA sequencing showed that primary tumor tissues, normal esophagus tissues, and PDOs formed separate clusters, reflecting the organoid culture environment compared to the heterogeneous cell type composition in the primary tumor specimen^39^. This highlights the issue that the microenvironment is missing in contemporary PDOs, which needs to be kept in mind when interpreting PDO-based research results.

The generation of PDAC PDOs was first reported by Boj et al.^9^. Three additional studies also focused on the generation of PDAC PDO libraries^45–47^. All studies identified common PDAC driver mutations in *KRAS*, *TP53*, *SMAD4*, and *CDKN2A* in PDOs. Interestingly, Tiriac et al. also observed uncommon genetic alterations in *ERBB2*, *PIK3CA*, and *MAP2K1*^45^. Alterations in *ERBB2* and *PIK3CA* led to marked sensitivity to afatinib or everolimus. Additionally, 70% of the PDOs showed characteristics of the classical or progenitor subtype, and ~30% showed characteristics of the basal or quasi-mesenchymal subtype. In contrast, most established monolayer 2D cell lines show expression profiles representative of the basal or quasi-mesenchymal subtype^53^. Seino et al. identified three functional PDAC subtypes with distinct WNT signaling dependencies by performing extensive genotype-phenotype correlation studies^46^.

The generation of LC PDOs was first reported by Broutier et al.^42^. Here, eight different organoid lines, including three hepatocellular cancer (HCC), three cholangiocellular carcinoma (CCC) and two combined HCC/CCC PDO lines, were analyzed by sequencing and shown to recapitulate the molecular profiles of the corresponding tumor of origin. The cultures also preserved the subtype-specific histology, gene expression profile, and mutational pattern of the parent tumor. Nuciforo et al. demonstrated that HCC/CCC organoids can be generated from ultrasound-guided needle biopsy samples^43^. Whole-exome sequencing results of the generated biopsy-derived PDO lines and primary tumor tissues did not significantly differ in terms of mutational rate, and typical frequent mutations of HCC/CCC in the driver genes *TP53*, *ARID1A*, and *TSC1* were found. However, the authors also identified several novel alterations in the PDOs that were not present in the original biopsies, indicating either detection problems in the biopsy sequencing or the preferred outgrowths of a subclone in PDO lines.

GEP-NENs are rare cancers occurring in the gastrointestinal tract with distinct histopathological features^54^. They are characterized by a loss of tubular structures and diffuse expression of neuroendocrine markers. Recently, the generation of a GEP-NEN organoid library was described by the Sato lab^48^. Altogether, 25 GEP-NEN PDOs were generated and analyzed by comprehensive molecular characterization. Whole-genome sequencing of GEP-NENs revealed frequent genetic alterations in *TP53* as well as *RB1* and characteristic chromosome-wide loss of heterozygosity.

Taken together, all these studies demonstrated that PDO libraries can be generated from the respective gastrointestinal cancer entities and that they molecularly resemble the tumor they were derived from and thus represent a powerful platform for studying cancer pathobiology in an ex vivo setting similar to the in vivo situation.

### Drug screening to delineate novel treatment strategies

The study of classical 2D cancer cell lines had provided major insights into drug responses and genetic alterations^55^. However, these 2D cancer cell lines poorly reflect the primary tumor tissue phenotypically and genetically, which may be the main reason for the high failure rate of newly developed drugs^2,56^. PDOs may better recapitulate primary tumors and might thus represent more accurate models for drug screening and discovery. To date, different drug screening approaches on living gastrointestinal PDO biobanks have been reported (Fig. 3b). The first medium-scale drug screen was described by the Clevers lab using their CRC organoid biobank^28^. Here, organoid cultures were plated in 384-well plates and treated with an 83-compound library with a wide range of cancer targets. The library was composed of 10 chemotherapeutics, 25 drugs in clinical use, 29 compounds currently in clinical trials, and 29 experimental compounds covering a diverse range of cancer targets. The study was the first to show a clear genotype-to-response correlation in PDOs for a number of alterations. For example, a mutation in the E3 ligase *RNF43* led to a sensitivity of CRC organoids to porcupine inhibitors, and PDOs with mutations in *TP53* were extremely resistant to the MDM2 inhibitor nutlin-3a, while *KRAS*-mutant organoids were resistant to ERBB inhibitors. This drug screen was the first to prove the feasibility of performing such an assay on PDOs, revealing the potential of PDOs to serve as clinically relevant biomarkers for therapy response testing. Vlachogiannis et al. screened metastatic CRC and GC/EAC PDOs with a medium-scale drug screen using a library consisting of 55 drugs currently in phase 1–3 clinical trials or in clinical practice^35^. Several genotype-drug phenotype correlations could be observed. As an example, one ERBB2-amplified PDO showed a good response to the dual ERBB2/EGFR inhibitor lapatinib, while treatment of an EGFR-amplified line with normal ERBB2 allele status had no effect.

GC PDOs were screened by Yan et al. in a medium-scale drug screen of 37 anticancer drugs in nine GC PDOs from seven patients^33^. The screen showed differential response to commonly used chemotherapeutics, with overall good response to oxaliplatin, epirubicin, and paclitaxel, while frequent resistance to 5-fluorouracil (5-FU) and oxaliplatin was seen. Additionally, the screen documented a sensitivity to targeted drugs, i.e., napabucasin (a STAT-3 inhibitor), abemaciclib (a CDK4/6 inhibitor), and VE-822 (an ATR inhibitor). Some of the drugs are approved or in clinical trials for disease entities other than GC, highlighting the potential of PDO biobanks in delineating potentially interesting cancer entities suitable for pharmaceutical company investments in clinical trials. A medium-scale drug screen in EAC PDOs with 24 anticancer compounds revealed resistance to classical chemotherapeutics, including 5-FU, epirubicin and cisplatin alone or in combination, but interestingly, the PDOs were sensitive to inhibitors of the receptor tyrosine kinases EGFR and ERBB2 and inhibitors of MEK1/2^39^.

LC PDOs were first screened by the Huch lab in a medium-scale drug screen with 29 anticancer compounds, including drugs in clinical use or development, which showed differential sensitivity for a subset of compounds^42^. For instance, treatment with the PI3K inhibitor taselisib resulted in a growth-inhibitory effect in five out of six LC PDOs. Additionally, ERK1/2 inhibition was proposed as a potential therapeutic approach for primary LC treatment. PDOs with insensitivity to MEK and BRAF inhibition showed an ambiguous response to ERK1/2 targeting. The lab of Selaru treated LC PDOs in a high-throughput drug screen with a library composed of 129 cancer drugs^44^. Overall, 13 drugs showed the ability to kill more than 90% of the LC PDOs, while nine of these drugs (HDAC, proteasome, DNA topoisomerase II, protein translation and RNA synthesis inhibitors) were effective for treating all PDOs.

The Tuveson lab analyzed the response of a PDAC organoid biobank to five chemotherapeutic drugs commonly used in PDAC treatment (gemcitabine, paclitaxel, irinotecan, 5-FU, and oxaliplatin)^45^. The reported data revealed that 33% of PDOs were resistant to all five drugs. Screening these multidrug-resistant lines with a targeted therapy panel of 21 drugs revealed a very high sensitivity to targeted agents for half of the PDOs. The authors concluded that drug screening of individual PDOs could reveal alternative treatment regimens when resistance to standard of care treatment is found. A second study by the Clevers lab assessed the feasibility of performing larger drug screens with PDAC organoids. PDAC PDOs were exposed to a drug library of 76 targeted therapeutics and classical chemotherapeutics^47^. For most PDOs, multiple compounds that resulted in effective tumor killing could be identified. However, no single therapy could be detected that worked for all PDOs, indicating the necessity to test each individual PDO to define the best treatment option.

Overall, the experimental setup for drug screen assays on PDO biobanks slightly differs between the different research groups. In general, drug response is analyzed by using a quantitative measure to assess viability after treatment. Organoids were plated in different plate formats (96- or 384-well plates) with different concentrations of Matrigel^®^ or BME^®^ (2–100%). Depending on the experimental setup, some studies exposed organoids to the treatments directly after seeding, e.g., using pre-spotted drug plates, while others studies allowed the organoids to recover for some days before the drugs were administered. This might result in differences in the effects of drugs on cell division and proliferation, as organoids show an increased proliferation rate after splitting, which decreases over time. One of the major barriers to screening PDO biobanks with medium- to large-scale drug libraries is the high cost of Matrigel^®^ or BME^®^. Reducing the required volume would directly affect this major matter of expense. Such miniaturization of the organoid platform for ultra-high-throughput drug screenings was reported by Du et al.^57^. Genetically engineered human colon organoids with a *KRAS*^*G12D*^ mutation were plated in 1536-well plates. For the assay, 2 µl BME^®^ per well was mixed with ~400 cells. Each well was covered with 5 µl growth medium, and for drug testing, 0.1 µl per drug was added. The comparison of the drug response in 1536-well plates to that in 384-well plates revealed identical growth inhibitory effects of the compounds. Due to miniaturization, drug library testing with 10,240 compounds (one concentration each) became feasible. Although the authors used a genetically modified colon organoid and not PDOs with a much higher variability in their growth as well as splitting behavior, the reported data demonstrate the potential for ultra-high-throughput assays using PDOs to define novel treatment options.

### Personalized medicine based on the testing of individual PDOs

**Fig. 4:**
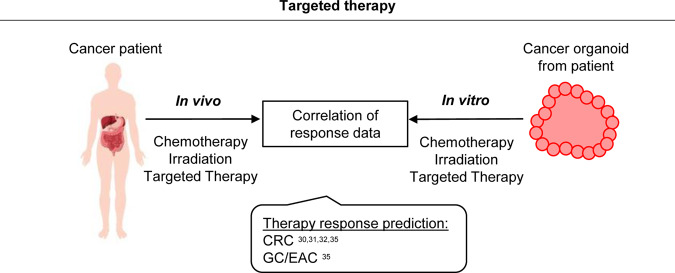
Personalized medicine based on the testing of individual PDOs. Different co-clinical studies have revealed the feasibility and potential use of PDOs as a predictive tool for therapy response testing. To this end, generated PDOs from cancer patients were treated in vitro with chemotherapy, irradiation or targeted therapy, and the obtained response data were correlated to the clinical responses of patients.

The main premise for personalized cancer therapy is the presence of patient-specific alterations and pathway aberrations depicting potential drug targets. Molecular profiling, drug assays, and targeted therapy testing on PDOs revealed promising results for different cancer entities. To date, different clinical studies have determined the feasibility and potential use of PDOs as a predictive test for therapy regimens (Fig. 4).

Vlachogiannis et al. were the first to report the potential of PDOs to predict therapy response using CRC and GC/EAC organoids generated from patients from four phase 1/2 clinical trials^35^. As an example, the response to anti-EGFR treatment with cetuximab was analyzed in five metastatic CRC PDOs and compared to the clinical response of the respective patients. Two PDOs established from biopsies before anti-EGFR treatment showed no response in line with the clinical response of the two patients. Both patients (unsurprisingly) had mutations in *KRAS*^*G12D*^ or *BRAF*^*V600E*^. A third cetuximab-resistant PDO line was established from a biopsy from a progressive tumor lesion that initially responded to anti-EGFR treatment. Molecular analysis revealed EGFR amplification, no RAS pathway mutations and high amphiregulin mRNA levels. These alterations theoretically should lead to responsive cancer cells, but treatment paradoxically led to increased proliferation of the respective PDO line. This finding highlights the potential of PDOs to predict clinical outcome better than molecular analyses alone at least in some cases. Overall, the authors reported an 88% positive predictive value of PDOs in forecasting the patient’s response to chemotherapy or target therapy. Ooft et al. additionally explored the power of PDOs derived from metastatic CRCs to predict the response to classical chemotherapy^32^. The study focused on regimens classically used in CRC treatment, including irinotecan as well as 5-FU/capecitabine alone or in combination with oxaliplatin or irinotecan. The authors could correctly predict the response for more than 80% of patients treated with irinotecan monotherapy or 5-FU/irinotecan combination therapy, while the response for the 5-FU/oxaliplatin combination therapy could not be predicted.

Ganesh et al. recently described the generation of a rectal cancer organoid biobank from tumor tissues of different stages and analyzed the ex vivo response of PDOs to clinically relevant chemotherapy and radiation treatment^30^. PDOs were treated with 5-FU alone or in combination with oxaliplatin. The obtained data revealed a diverse spectrum of dose responses, and the response predictions were correlated with the clinical outcomes of the patients. Furthermore, the authors tested radiation treatment, which also resulted in heterogeneous responses among PDOs. The displayed sensitivity was consistent with the patients’ clinical outcomes upon irradiation. As part of a clinical phase III trial, Yao et al. reported a locally advanced rectal cancer organoid biobank^31^. In this study, PDOs were exposed to 5-FU or irinotecan chemotherapy or irradiation. Different PDO lines showed diverse responses to individual treatments. The authors showed that the patients achieved a good clinical response when their corresponding PDOs were sensitive to at least one of the three treatments. Furthermore, combinatorial treatment also resulted in consistent outcomes between the PDOs and the patients. All in all, the authors could predict the therapy response in 85% of patients, illustrating the potential of rectal cancer PDOs to stratify rectal cancer patients correctly as responders and non-responders.

To date, several co-clinical studies have already suggested a high accuracy of PDOs to correctly predict therapy response. It will now be important to optimize protocols with regard to a high culturing rate and fast expansion to allow timely drug testing. This will allow the integration of PDOs into clinical settings, which is the prerequisite to validating these promising preliminary results in prospective clinical trials.

### Investigation of intratumoral heterogeneity and tumor evolution

**Fig. 5:**
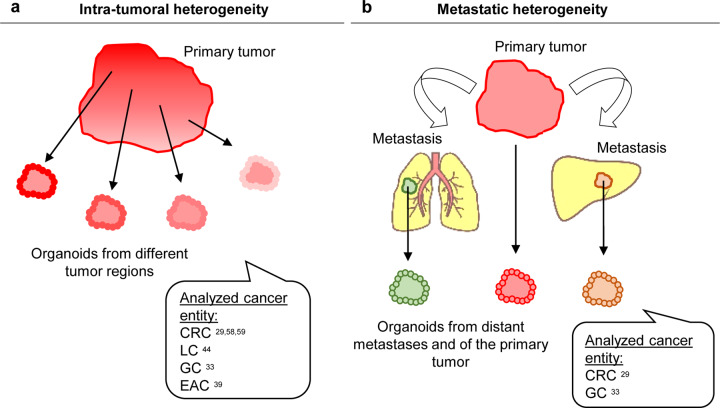
Investigation of intratumoral heterogeneity and tumor evolution. **a** PDOs provide an excellent platform to study the functional relevance of intratumoral heterogeneity by generating organoids from different regions of the tumor. **b** Cancer organoids have also been used to study tumor evolution by comparing PDOs from primary and metastatic sites of a patient.

Gastrointestinal cancers have been shown to contain to varying degrees different subclones that differ genetically from each other. PDOs now provide an excellent platform to study the functional relevance of intratumoral heterogeneity, i.e., if genetic differences have an impact on the biological behavior and drug response (Fig. 5a). Fujii et al. generated CRC organoids from different tumor regions of the same patient^29^. Cancer organoids from a primary tumor with histopathological heterogeneity resulted in an organoid culture with distinct morphologies, which could be isolated and subsequently grown independently. The majority of driver mutations were conserved between the subcloned lines, except for a *TP53* mutation, which was unique to one line. Roerink et al. reported the generation of clonal CRC organoids from multiple single cells of different tumor regions of three CRC patients and adjacent normal intestinal crypts^58^. As expected, cancer cells showed extensive molecular diversification and carried a higher mutation burden than normal cells. Whole-genome sequencing allowed the generation of phylogenetic trees of tumor evolution for each patient. These phylogenetic trees consisted of a trunk containing mutations common to all clonal CRC cells from one patient that was then divided into subsequent trees, with the ultimate tree containing individual mutations of an individual clonal organoid line. In general, clonal organoids from one region clustered together and shared common driver mutations, but individual organoid lines from one region still exhibited substantial differences in their mutational pattern (up to 40% of mutations were not shared). Most interestingly, molecular diversification translated into differential responses upon drug treatments. Of note, even within clonal organoid lines of one tumor region, substantial differences were observed for classical chemotherapeutics and targeted therapies. In line with this, a further study by Schumacher et al. investigated the impact of intratumoral heterogeneity on treatment effects in CRC^59^. The authors generated five sibling organoid cultures from separate regions of one patient and showed that the sibling cultures exhibited remarkable genetic heterogeneity and did not respond uniformly to targeted therapies.

Intratumoral heterogeneity can also be found in LC and GC/EAC and resolved in PDO cultures. LC PDOs derived from distinct tumor regions of five patients were assessed by drug screening, revealing pan-, inter- and intra-patient effective drugs^44^. Comparisons of PDOs from different regions of GC specimens revealed marked differences in the aberration patterns^33^. While key driver genes such as *TP53*, *CDH1*, and *ARID1A* were again common to both tumor regions, six out of nine PDOs showed subclonal mutations, including mutations in genes such as *APC* and *BRCA2*, that were only present in one of the two analyzed regional PDOs. Similar to CRC, differential responses of regional PDOs to drug treatments were observed. Li et al. did not analyze different tumor regions but instead used spectral karyotyping to reveal the clonal hierarchy and dynamics in EAC PDOs^39^. The data revealed that most PDO cells were aneuploid, and all cultures consisted of heterogeneous cell populations with a variety of chromosome numbers and chromosomal rearrangements. To investigate clonal dynamics, whole-genome sequencing was performed at different time points up to 6 months. These analyses revealed that during the process of establishing PDOs, selection pressure exists that selects genetic subclones but that organoid heterogeneity is maintained during subsequent culturing. Interestingly, the dynamic clonal behavior varied between the lines, from relatively stable to ongoing in others.

Cancer organoids have also been used to study tumor evolution by comparing PDOs from primary and metastatic sites of a patient (Fig. 5b). As an example, Fujii et al. generated four independent matched sets of primary CRC and metastatic lesions^29^. Exome sequencing confirmed common driver mutations between the primary and metastatic couples. Interestingly, the metastasis-derived organoids showed a higher metastatic capacity upon xenotransplantation, indicating that PDOs of different sites (primary vs. metastatic) functionally differ while harboring similar aberrations. Yan et al. analyzed paired organoid cultures of primary tumors and lymph node metastases of GC, detecting varying degrees of heterogeneity from couple to couple^33^. PDOs from primary tumors and lymph node metastases of diffuse-type GC showed only a few differences. However, marked differences were recognized in mixed-type GC, as only 23% of mutations were shared between the primary tumor and metastasis. Histologically, the primary tumor showed a diffuse growth pattern, while lymph node metastases had a glandular morphology.

Overall, the performed studies indicate that PDOs from different tumor regions and from different sites of metastatic spread exhibit similarities in molecular features, e.g., in driver gene mutations, but vary strikingly in terms of their drug responses or metastatic capability. This emphasizes the value of PDOs as a “living system”, allowing researchers to perform functional assays such as drug tests and metastasis assays to reveal important differences.

### Studying the tumor microenvironment with PDOs

**Fig. 6:**
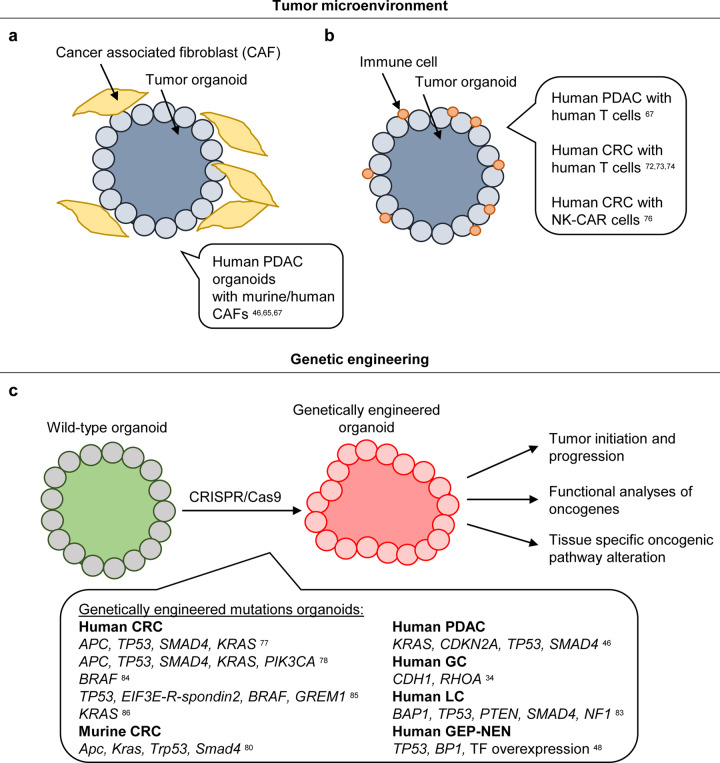
Studying the tumor microenvironment and engineering of tumor organoids. **a** To analyze the interaction between cancer cells and the tumor microenvironment, gastrointestinal tumor organoids were co-cultivated with cancer-associated fibroblasts (CAFs). **b** To analyze the interaction between immune cells and malignant cells, to study the interaction and to allow the testing of immunotherapeutic approaches, gastrointestinal tumor organoids were co-cultivated with immune cells. **c** Wild-type organoids can be engineered into tumor organoids using CRISPR/Cas9 by generating tumorigenic mutations to study tissue-specific oncogenic pathway alterations, to monitor cancer initiation and progression and to functionally analyze (candidate) oncogenes.

Tumors grow in a complex environment consisting of the ECM and non-malignant stromal cells, including fibroblasts, endothelial cells, and immune cells^60–62^. The tumor microenvironment plays an essential role in tumor development and progression, as bi-directional signaling between tumor and environment fosters tumor growth. For example, cancer-associated fibroblasts (CAFs) have the potential to secrete ECM molecules and soluble factors that can further stimulate tumor growth, cell survival, and metastasis^63,64^. Nevertheless, the positive impact of the microenvironment on tumor growth or therapy resistance provides novel therapeutic points of interference. To date, the interaction of the microenvironment with tumor cells has mainly been studied only in vivo. Nevertheless, there is a need to study the interactions in vitro in a controlled environment enabling direct visualization. However, cancer organoids typically consist of pure tumor cells with no additional stroma or immune cells. Fibroblasts are usually observed in the initial organoid culture after plating but are lost in early passages of culturing. To circumvent this limitation, the Tuveson lab used a co-cultivation approach of human PDAC organoids and murine CAFs (Fig. 6a)^65^. This led to the activation of fibroblasts and further production of desmoplastic stroma. This system resulted in the formation of two distinct fibroblast types: tumor-proximal myofibroblasts and tumor-distal fibroblasts expressing inflammatory markers. This heterogeneity within CAFs has therapeutic implications, as some CAF populations have anti-tumorigenic features and should not be targeted but instead should be selectively stimulated.

Seino et al. also described a co-cultivation approach for human PDAC organoids with CAFs^46^. The authors reported in their PDAC biobank three functionally different subtypes based on their dependency on the stem cell niche factors WNT and Rspondin. One of these subtypes is the non-WNT-producing subtype, which critically depends on exogenous WNT in the medium. Interestingly, when these WNT-dependent PDAC organoids were co-cultivated with patient-derived CAFs, their growth became independent of exogenous WNT. Of note, the physical proximity of CAFs to organoids was necessary to support growth. Overall, the established co-culture system proved that patient-derived CAFs provide the niche by secreting WNT for this non-WNT-producing subtype. The drug resistance of human tumors to anticancer drugs is often related to gene mutations, transcriptomic and epigenetic changes and to the tumor microenvironment^66^. Tsai et al. described that the co-cultivation of human PDAC PDOs and human CAFs increased resistance to gemcitabine treatment^67^. This finding confirms the notion that the tumor microenvironment can mediate therapy resistance, which can be functionally tested in PDO CAF co-culture assays.

Since its initial success in the clinic, cancer immunotherapy has become the rising star of cancer therapy in recent years^68–70^. Immune effector cells (e.g., T lymphocytes and natural killer (NK) cells) detect the presence of tumor-associated antigens expressed on tumor cells, which can then be killed by immune effector cells. Similar to what has been discussed above, observing the process of activation of the immune system followed by cytotoxic killing of malignant cells in vitro would greatly help to understand the biology behind and allow functional testing of the effectiveness of immunotherapeutics for a certain patient. Recent studies have successfully activated immune cells by co-cultivation with PDOs, which resulted in a killing cancer organoids (Fig. 6b). As an example, Finnberg et al. demonstrated that tumor organoids can be co-cultured for up to eight days together with CD45^+^ lymphocytes in an ALI system^71^. Tsai et al. published a model of tumor-immune signaling in which T lymphocytes isolated from blood were cultivated in culture medium on top of Matrigel^®^ containing human PDAC or normal pancreatic organoids^67^. Interestingly, T cells invaded the ECM surrounding the cancer organoids, which was not seen in co-cultures with normal organoids. A different co-cultivation approach was described by Dijkstra et al., who used circulating tumor reactive T lymphocytes together with human CRC organoids^72,73^. To generate tumor reactive T cells, human peripheral blood lymphocytes were isolated and co-cultured repetitively with interferon γ pre-stimulated CRC organoids (to enhance antigen presentation to T lymphocytes). The co-culture led to antigen-specific stimulation and activation of T cells. Two applications of this co-culture method can be foreseen. First, the potential effect of immunotherapeutics could be evaluated in vitro. Second, patient-specific T cells can be generated and expanded for adoptive T cell transfer. Another study assessed the functionality of tumor-infiltrating T lymphocytes upon chemoradiotherapy in CRC organoids^74^. In a proof-of-principle study, rectal cancer organoids and infiltrating T lymphocytes were separately expanded, co-cultured and analyzed for cytotoxic effects. The killing effects of T cells were significantly greater for organoids from patients who responded well to chemoradiotherapy than for organoids from non-responders. The additional blockage of PD-1 on T lymphocytes with increased PDL-1 expression led to a recovery of antitumor immunity against PDOs in some patients.

Immunotherapies using chimeric antigen receptor (CAR) lymphocytes have led to impressive responses in various hematological malignancies, and the use of CAR-engineered T cells for leukemia therapy was recently approved by the FDA^75^. The application of CAR T cells in solid cancers is more challenging due to the limited number of expressed neoantigens, among other reasons. Nevertheless, in a proof of concept study, the Farin laboratory established a platform to test CAR cell cytotoxicity toward PDOs^76^. Using an NK cell line in which CAR was introduced to target the ubiquitously expressed epithelial antigen EPCAM, CAR-mediated toxicity could be analyzed in CRC as well as in healthy organoids. Next, NK-CAR cells targeting the tumor-specific neoantigen epidermal growth factor receptor variant III (EGFRvIII) were used in conjunction with an EGFRvIII-expressing CRC PDO, resulting in efficient killing of the PDO, while normal organoids remained unharmed. The results demonstrated the usefulness of organoid CAR platforms for assessing tumor cell specificity.

The presented studies demonstrate the potential applications of co-cultivation assays of PDOs from different malignant tumors combined with stromal, immune, or CAR cells in the discovery and development of novel therapeutic strategies that will target stromal compartments or help to direct immune cells to their target.

### Cancer modeling by genetic engineering of healthy tissue-derived organoids

Organoids derived from cancers are unique in their genetic makeup. Each PDO is an avatar of the cancer it is derived from, making it an ideal tool to study individual therapeutic strategies or to test new substances in large PDO biobanks to find the ideal cancer entity for further clinical studies. Nevertheless, when general biological questions are the focus of research, genetically defined models are necessary. The genetic manipulation of healthy tissue-derived human organoids, e.g., by CRISPR/Cas9, enables the generation of such defined models. Such models are ideal tools to study, among others, initial malignant transformation, the effect of certain altered signaling pathways on the tissue of interest or the functional analysis of putative oncogenic candidate genes (Fig. 6c).

The first organoid model with a defined genetic setup was generated using ALI technology from murine pancreatic, gastric and colonic tissue^21^. The Kuo laboratory generated mouse lines combining up to four floxed alleles of *Apc*, *Trp53*, *Kras*^*G12D*^, and *Smad4*, which were recombined by Cre in vitro and subsequently analyzed^21^. While pancreatic and gastric ALI organoids displayed dysplasia in vitro, quadruple-mutant colon ALI organoids could be transformed to adenocarcinomas. Starting from healthy intestinal organoids, two studies reported the use of CRISPR/Cas9 genome editing technology to introduce different combinations of common CRC driver mutations to dissect cancer progression. Drost et al. from the Clevers lab sequentially mutated the tumor suppressor genes *APC*, *TP53*, and S*MAD4* and added G12D activation of the *KRAS* oncogene to generate quadruple-mutant organoids^77^. Matano et al. from the Sato lab in parallel generated the same quadruple-mutant organoids and added an activating mutation in the *PIK3CA* gene^78^. Both studies showed that these driver mutations resulted in tumor growth independently of all human intestinal stem cell niche factors by growing them in medium without EGF, WNT, Rspondin, and Noggin. After subcutaneous xenotransplantation into mice, the quadruple-mutant tumor organoids from the Clevers lab formed tumors with invasive features without metastatic spread. Of note, orthotopic implantation of the quadruple-mutant tumor organoids, but not any kind of combination of triple-mutant organoids, into the cecum of mice led to spontaneous metastatic spread into the lung and liver^79^. Interestingly, when the Sato lab engineered triple-mutant organoids starting from chromosomally unstable human adenomas, splenic injection resulted in micrometastases in the liver^78^. The de Sauvage laboratory modeled the quadruple-mutant model with the combination of *Apc*, *Kras*, *Trp53*, and *Smad4* changes in murine small intestinal organoids, combined Cre/Lox with CRISPR/Cas9 technology, and transplanted these organoids orthotopically into mice^80^. Invasive CRC carcinoma with metastatic spread was observed, but most interestingly, simultaneous depletion of Lgr5-positive cancer stem cells led to a substantial decrease in liver metastatic burden. Two additional studies underline the need for an orthotopic transplantation environment of engineered organoids to study CRC metastasis^81,82^. Taken together, several studies thus indicate that in addition to the genetic composition of the engineered tumor organoids, the site of transplantation and therefore the local environmental conditions are also important.

In addition to CRC, CRISPR/Cas9 technology has also been used to model other gastrointestinal cancer entities. For example, the knockout of *TP53* and *BP1* combined with the overexpression of key transcription factors in normal colonic epithelium led to a GEP-NEN organoid phenotype with neuroendocrine marker expression, a high KI67 index and a solid morphology^48^. Seino et al. reported the in vitro generation of PDAC organoids by combining a *KRAS*^*G12V*^ mutation and loss-of-function mutations in *CDKN2A*, *TP53*, and *SMAD4* starting from normal human pancreas organoids^46^. The quadruple-mutant organoids showed histological transformation into cancer cells with features ranging from benign lesions to invasive PDACs after subcutaneous xenotransplantation. Nanki et al. modeled the impact of two frequently co-occurring mutations in diffuse gastric cancer (*CDH1*, encoding the cell–cell junction protein E-cadherin, and *RHOA*, a member of the Rho family of small GTPases) in normal human gastric organoids^34^. The loss of *CDH1* led to a phenotypical change from normal cystic structures to a grape-like morphology and induced migratory activity. Nevertheless, without additional inhibition of RHOA-ROCK-induced cell death either via ROCK inhibitor treatment or via generation of a compound mutant line with *RHOA* knockout, the *CDH1* single knockout organoids diminished over time. This study intriguingly demonstrated the power of combining organoid technology with genome editing techniques. Artegiani et al. used normal human cholangiocyte organoids and knocked out the tumor suppressor BAP1, resulting in impaired chromatin accessibility, a loss of multiple epithelial characteristics and an increase in motility^83^. The combination of common cholangiocarcinoma mutations (*TP53*, *PTEN*, *SMAD4*, *NF1*) concomitant with *BAP1* resulted in the acquisition of tumorigenic features after subcutaneous or intrahepatic injection^83^.

In addition to generating organoid models with multiple alterations culminating in advanced carcinomas, CRISPR/Cas9 technology can be used to study the early tumorigenesis of tumor subtypes. For example, the Medema lab analyzed the response of two distinct CRC precursor lesions, tubular adenomas (TA), and sessile serrated adenomas (SSA), to TGFβ treatment^84^. While TA organoids can be easily cultured, SSA cells do not grow as organoids. Therefore, the prototypic mutation of SSAs, the *BRAF*^*V600E*^ mutation, was genetically engineered in normal colon organoids. While TA organoids responded with apoptosis to TGFβ treatment, a mesenchymal phenotype was induced in SSA organoids. The authors conclude that the activation of the RAS pathway by *BRAF*^*V600E*^ combined with TGFβ stimulation leads to the development of the mesenchymal subtype of CRCs. Interestingly, SSAs are not only characterized by *BRAF*^*V600E*^ mutations but also show recurrent Rspondin gene fusions. Therefore, Kawasaki et al. studied the effect of Rspondin fusions in human colon organoids by generating chromosomal rearrangements for the first time using the CRISPR/Cas9 system^85^. Chromosomal rearrangements, including deletions and inversions affecting Rspondin2, were introduced into *TP53*-disrupted human colon organoids. Additionally, *BRAF*^*V600E*^ mutations were inserted, and the TGFβ inhibitor gremlin (*GREM1*) was overexpressed. Xenografted tumors originating from triple-mutant organoids formed flat serrated lesions similar to SSAs. This study impressively shows that CRISPR/Cas9 technology can not only be used to mutate genes but can also be used to induce chromosomal fusion generated by deletion or inversion as well as interchromosomal translocation. CRISPR/Cas9 can also be used to delineate treatment strategies by specifically altering a certain pathway. Verissimo et al. studied the EGFR/MEK/ERK pathway in CRC organoids and introduced the oncogenic *KRAS*^*G12D*^ mutation in CRC organoids with *APC* and *TP53* alterations^86^. The effect of *KRAS* was analyzed in single or combinational therapies using the dual EGFR/HER2 inhibitor afatinib and the MEK inhibitor selumetinib. No response to single-agent treatment was seen in the genetically modified organoids, while sensitivity to the combination treatment was observed. In conclusion, CRISPR/Cas9 technology provides an excellent experimental platform for mechanistic studies on cancer genes and pathways in a human context.

## Future prospects and conclusion

Organoid cultures of various cancers revealed to have a great impact on cancer research with widespread usage in basic and translational cancer research. Individual PDOs constitute avatars of patients’ tumors, which might help clinicians tailor treatment strategies on an individual level by predicting the response to anticancer drugs. Collections of PDOs, constituting living biobanks containing a broad spectrum of different molecular and histological subtypes of a certain cancer entity, are thought to help with identifying patient subgroups that should respond to newly developed drugs or repurposing existing drugs. However, some cancer entities (e.g., sarcomas) do not regularly form organoids, and the frequency of successful organoid outgrowth for some entities is far from being close to 80–90%, indicating that a relevant subtype exists that is in need of special growth conditions. Thus, new protocols and the continuous improvement of existing culturing methodologies are needed. The culturing costs are substantial, and the reagents and culture conditions differ among laboratories, so a consensus is needed. The presence of serum in conditioned medium used for some growth factors (e.g., WNT) prohibits the use of organoids in the clinical setting, but the problem might be bypassed with artificial WNT3A agonists^87^ or afamin-stabilized WNT3A^88^. The greatest current challenge in organoid culture is certainly the absence of the microenvironment. Nevertheless, the field is advancing rapidly, and protocols are developed that allow the incorporation of cells from the tumor microenvironment, such as fibroblasts and immune cells. Genomic, transcriptomic, and proteomic analyses of human cancer organoids help to better understand tumor biology and define targeted therapies, and the treatment of organoids allows direct assessment of the response to the identified drugs. The first co-clinical trials of patients and PDOs treated side-by-side have shown the power of organoids in predicting the treatment response. If these results are confirmed in prospective clinical trials, PDOs could ultimately be involved in clinical decision making. In conclusion, cancer organoids might bridge the gap between molecular genetics, the current biological understanding and clinical therapy in the near future.
